# RETRACTED: Chae et al. Anticancer Activity of 2-*O*-Caffeoyl Alphitolic Acid Extracted from the Lichen, *Usnea barbata* 2017-KL-10. *Molecules* 2021, *26*, 3937

**DOI:** 10.3390/molecules30244664

**Published:** 2025-12-05

**Authors:** Hae-Jung Chae, Geum-Jin Kim, Barsha Deshar, Hyun-Jin Kim, Min-Ji Shin, Hyukbean Kwon, Ui-Joung Youn, Joo-Won Nam, Sung-Hak Kim, Hyukjae Choi, Sung-Suk Suh

**Affiliations:** 1 Department of Bioscience, Mokpo National University, Muan-gun 58554, Jeonnam, Republic of Korea; 0o_hj_4004@naver.com (H.-J.C.); barsha.deshar.97@gmail.com (B.D.); minji4099@naver.com (M.-J.S.); 2 College of Pharmacy, Yeungnam University, Gyeongsan-si 38541, Gyeongsangbukdo, Republic of Korea; canta87@ynu.ac.kr (G.-J.K.); zero9602@gmail.com (H.K.); jwnam@yu.ac.kr (J.-W.N.); 3 Research Institute of Cell Culture, Yeungnam University, Gyeongsan-si 38541, Gyeongsangbukdo, Republic of Korea; 4 Department of Animal Science, College of Agriculture and Life Sciences, Chonnam National University, Gwangju 61186, Republic of Korea; redallert0@gmail.com (H.-J.K.); sunghakkim@jnu.ac.kr (S.-H.K.); 5 Department of Biomedicine, Health & Life Convergence Science, BK21 Four, Mokpo National University, Muan-gun 58554, Jeonnam, Republic of Korea; 6 Division of Life Sciences, Korean Polar Research Institute (KOPRI), Incheon 21990, Republic of Korea; ujyoun@kopri.re.kr; 7 Department of Polar Sciences, University of Science and Technology, Incheon 21990, Republic of Korea

The journal retracts the article titled “Anticancer Activity of 2-*O*-Caffeoyl Alphitolic Acid Extracted from the Lichen, *Usnea barbata* 2017-KL-10” [1], cited above.

Following publication, concerns were brought to the attention of the Editorial Office regarding the integrity of a number of images presented in this publication [1].

Adhering to our standard procedure, the Editorial Office and Editorial Board conducted an investigation that confirmed indications of inappropriate editing in Figure 5 and partial duplication between Figures 5 and 6 in the cited article [1] and Figures 5B and 6B from an earlier publication [2] presenting different experimental conditions. While the authors collaborated within this process, the full raw material meeting the journal’s requirements for original images could not be provided for Editorial Board evaluation.

Consequently, the Editorial Board has lost confidence in the reliability of the findings and has decided to retract this publication as per MDPI’s retraction policy (https://www.mdpi.com/ethics#_bookmark30).

This retraction was approved by the Editor-in-Chief of *Molecules*.

The authors Sung-Suk Suh, Hyun-Jin Kim, Hyukjae Choi, Barsha Deshar, and Min-Ji Shin agree with the retraction. The rest of the authors did not provide a comment on this decision.
